# One-Carbon Metabolism Associated Vulnerabilities in Glioblastoma: A Review

**DOI:** 10.3390/cancers13123067

**Published:** 2021-06-19

**Authors:** Kimia Ghannad-Zadeh, Sunit Das

**Affiliations:** 1 The Arthur and Sonia Labatt Brain Tumour Research Centre, The Hospital for Sick Children, Toronto, ON M5G 0A4, Canada; Kimia.ghannad.zadeh@mail.utoronto.ca; 2 Institute of Medical Science, University of Toronto, Toronto, ON M5S 1A8, Canada; 3 Division of Neurosurgery, St. Michael’s Hospital, Toronto, ON M5B 1W8, Canada

**Keywords:** glioblastoma, glioma, one-carbon metabolism, de novo purine synthesis, metabolic reprogramming, metabolic treatment

## Abstract

**Simple Summary:**

Glioblastoma tumours are the most malignant and common type of central nervous system tumours. Despite aggressive treatment measures, disease recurrence in patients with glioblastoma is inevitable and survival rates remain low. Glioblastoma cells, like other cancer cells, can leverage metabolic pathways to increase their rate of proliferation, maintain self-renewal, and develop treatment resistance. Furthermore, many of the metabolic strategies employed by cancer cells are similar to those employed by stem cells in order to maintain self-renewal and proliferation. One-carbon metabolism and de novo purine synthesis are metabolic pathways that are essential for biosynthesis of macromolecules and have been found to be essential for tumourigenesis. In this review, we summarize the evidence showing the significance of 1-C-mediated de novo purine synthesis in glioblastoma cell proliferation and tumourigenesis, as well as evidence suggesting the effectiveness of targeting this metabolic pathway as a therapeutic modality.

**Abstract:**

Altered cell metabolism is a hallmark of cancer cell biology, and the adaptive metabolic strategies of cancer cells have been of recent interest to many groups. Metabolic reprogramming has been identified as a critical step in glial cell transformation, and the use of antimetabolites against glioblastoma has been investigated. One-carbon (1-C) metabolism and its associated biosynthetic pathways, particularly purine nucleotide synthesis, are critical for rapid proliferation and are altered in many cancers. Purine metabolism has also been identified as essential for glioma tumourigenesis. Additionally, alterations of 1-C-mediated purine synthesis have been identified as commonly present in brain tumour initiating cells (BTICs) and could serve as a phenotypic marker of cells responsible for tumour recurrence. Further research is required to elucidate mechanisms through which metabolic vulnerabilities may arise in BTICs and potential ways to therapeutically target these metabolic processes. This review aims to summarize the role of 1-C metabolism-associated vulnerabilities in glioblastoma tumourigenesis and progression and investigate the therapeutic potential of targeting this pathway in conjunction with other treatment strategies.

## 1. Introduction

Altered cell metabolism is a hallmark of cancer cell biology [1]. Many groups have identified ways in which cancer cells use adaptive metabolic strategies to facilitate the process of tumourigenesis. Folate-mediated one-carbon (1-C) metabolism is a metabolic process in which 1-C unit carriers are produced for use in biosynthetic pathways [2]. Recently, there has been great interest in the role of 1-C metabolism in cancer cell proliferation with many genomic and metabolomic studies showing upregulation of this metabolic process in various cancers, including glioblastoma [2,3].

Glioblastoma is the most common primary brain tumour in adults [4]. Despite aggressive treatment, including resective surgery followed by concomitant radiotherapy and chemotherapy, treatment failure and disease recurrence remain universal [4,5]. The constant nature of recurrence in glioblastoma and general ineffectiveness of second line therapies highlight the need for improved understanding of the molecular characteristics of this disease and the development of novel approaches to its treatment.

Reprogramming of cellular metabolism has been identified as a critical step in glial cell transformation during glioblastoma tumourigenesis [6]. Metabolic reprogramming in glioma cells has been studied in the context of a variety of mechanisms, including increased Warburg effect and aerobic glycolysis [7,8,9,10], the pentose phosphate pathway (PPP) [11,12,13,14,15], amino acid metabolism [16,17,18,19], oxidative phosphorylation [14,20,21,22,23,24], and lipid metabolism [25,26,27,28,29,30]. Many of these metabolic pathways manifest in synthesis of macromolecules needed for proliferation.

Among the various metabolic strategies used by glioma cells, the folate-methionine pathway and 1-C metabolism remain understudied [7]. These metabolic pathways are critical for nucleotide synthesis and DNA methylation [2,7,11,31]. Additionally, de novo purine synthesis and upregulation of the related 1-C metabolism pathway have been noted as characteristics of less differentiated stem and progenitor cells as well as brain tumour initiating cells (BTICs) responsible for tumorigenesis [31,32,33,34]. In the following sections, we aim to summarize the role of 1-C metabolism-associated vulnerabilities in cancer, and particularly in glioma cells. Additionally, we will evaluate whether this altered metabolic program can serve as a phenotypic identifier of BTICs and as a potential therapeutic target in glioblastoma. Further elucidation of the role of 1-C metabolism-related vulnerabilities in glioblastoma might uncover novel mechanisms that mediate and control cell proliferation and reveal effective novel treatment strategies.

## 2. Metabolic Reprogramming in Cancer and Cancer Initiating Cells

Tumourigenic cells alter their metabolic processes to meet the increased substrate demands required to sustain rapid proliferation, self-replication, and invasion. Since the identification of the Warburg effect, many groups have identified a variety of ways in which cancer cells reprogram metabolic pathways. In fact, metabolic reprogramming has been established as one of the hallmarks of cancer [1,9]. Metabolic programs play a significant role in balancing proliferation and cell-fate regulation. This role becomes particularly important in stem cells, which need to retain self-renewal capacity and the ability to differentiate [35]. Interestingly, cancer cells and normal stem cells share a number of similarities in their signalling pathways regulating metabolic phenotypes, which are conducive to increased proliferation, enhanced self-renewal, and improved adaptability to differing environmental conditions [35].

The first metabolic alteration in cancer cells was observed to be an upregulation in glucose uptake and a preference for glycolysis in oxygen-rich environments, a phenomenon referred to as aerobic glycolysis, or the Warburg effect [9]. Cancer cells and stem cells both engage in increased levels of aerobic glycolysis [36,37]. Additionally, both cancer cells and stem cells are heavily reliant on exogenous glucose and glutamine supplies [38,39,40].

Upstream of the mentioned metabolic changes, cancer cells and stem cells share a number of growth signalling pathways involved in metabolic regulation. In normal cells, growth factor-mediated activation of receptor tyrosine kinases engages signalling pathways such as PI3K, Ras, MEK/ERK, and mTOR to increase anabolic pathways and macromolecule synthesis [41]. These pathways are often overactivated in cancer cells, and many have also been shown to regulate pluripotent cell growth [35].

A number of the discussed metabolic alterations have been reported in connection with pro-oncogenic signalling in glioma cells [7]. In glioblastoma cell lines, activation of ERK1/2 by epithelial growth factor (EGF) leads to the nuclear translocation of pyruvate kinase M2 (PKM2), a critical enzyme involved in the production of pyruvate in the glycolysis pathway, leading to a positive feedback loop that ultimately results in an increase in aerobic glycolysis [42]. The PPP, which is necessary for the maintenance of a constant supply of nucleotides, has been shown to be upregulated in actively dividing cells within gliomas [43]. Mutations in Krebs cycle enzymes isocitrate dehydrogenase 1/2 (IDH1/2) are present in a subset of glioblastoma cases, affecting amino acid metabolism and glucose oxidation [16]. Our group has shown that the reduction in glioblastoma tumour formation after inhibition of inhibitor of DNA-binding 1 (ID1) is mediated by downregulation of EGF and downstream ERK1/2 signalling [44]. ERK1/2 activation induces transcriptional regulators of glycolysis, the tricarboxylic acid cycle, and macromolecular biosynthesis, as well as cell proliferation programs [8]. Furthermore, ID1 is a marker of relatively quiescent glioma stem-like cells that are required for tumourigenesis, are resistant to chemotherapy, and can be responsible for initiating tumour recurrence [44,45]. These data suggest that metabolic reprogramming may play a role in mediation of the stem-like phenotype in glioma cells.

Cancer stem cells are a class of cells that exhibit the features of both normal stem cells and cancer cells; however, the metabolic characteristics of these cells, especially BTICs, have been poorly understood [35,46]. It has been suggested that BTICs are less glycolytic than more differentiated glioma cell populations [47]. Additionally, BTICs are known to have increased glucose uptake and upregulation of the de novo purine synthesis pathway, metabolic pathways which allow maintenance of rapid proliferation and growth [32]. Further, BTICs have a higher mitochondrial reserve than differentiated glioma cells, suggesting that these cells use adaptive metabolic strategies to resist therapeutic stress [47]. These data suggest that metabolic alterations, particularly in certain pathways such as nucleotide synthesis, may be a characteristic of the stem-like phenotype in glioma and may thus be critical to treatment resistance.

## 3. 1-C-Mediated de Novo Purine Synthesis: A Brief Overview

The abundance of the nucleotide pool, as well as the level and activity of different rate-limiting enzymes of the nucleotide synthesis pathway, significantly affects the proliferative capacity of cells as well as their capacity for DNA replication and repair [15]. 1-C metabolism and the closely related purine synthesis pathway are critical to these issues [7].

1-C metabolism provides carbon units for biosynthesis through folate intermediates. Tetrahydrofolate (THF), after entering the 1-C cycle, can bind methyl groups and act as a carbon donor. 10-Formyl-THF is produced in the mitochondria from the reduction of 5,10-methyl-THF by methylenetetrahydrofolate dehydrogenase 2-like protein (MTHFD2/L), and is primarily involved in de novo purine synthesis [2,48]. Cells require a steady supply of nucleotides to complete the processes of DNA replication and cell division. Nucleotides can be produced either through salvage pathways recycling existing nucleobases or through de novo synthesis pathways [49]. De novo purine synthesis has the largest demand for 1-C units [2]. De novo purine synthesis results in the production of inosine monophosphate (IMP) from phosphoribosyl pyrophosphate (PRPP), which is further converted into guanosine monophosphate (GMP) or adenosine monophosphate (AMP). De novo purine synthesis is preferentially activated in conditions with higher requirement for purine nucleotides, such as in rapidly dividing cells [49,50,51,52]. The reactions of de novo purine synthesis are mediated in the cytosol by enzymes working in a metabolic complex named the purinosome, increasing the efficiency of this anabolic process [53,54].

**Figure 1:**
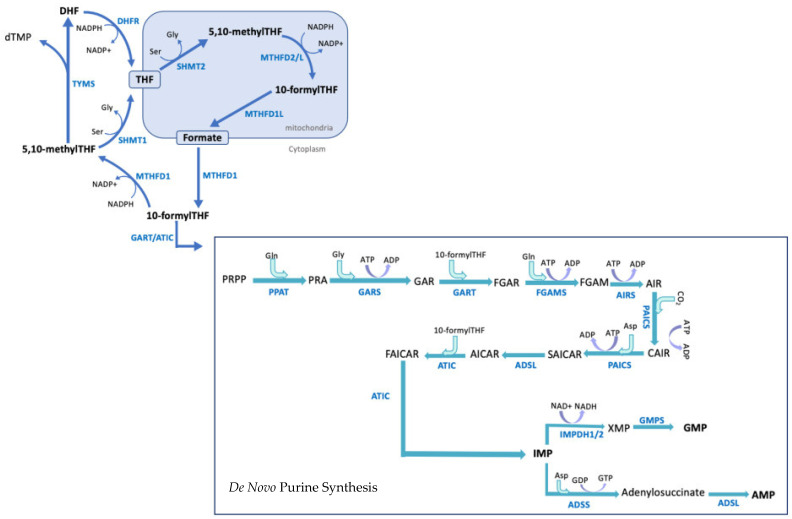
One-carbon-mediated de novo purine synthesis. Dietary folate is reduced to dihydrofolate (DHF) and subsequently tetrahydrofolate (THF) by dihydrofolate reductase (DHFR). THF is acted on by a series of enzymes in the mitochondria, which add methyl groups to THF, allowing it to act as the initial 1-C carrier required for a variety of biosynthesis processes. 10-Formyl-THF is produced in the mitochondria from the reduction of 5,10-methyl-THF by methylene tetrahydrofolate dehydrogenase 2 (MHFD2/L). 10-Formyl-THF is then used in de novo purine synthesis as a carbon donor. The purine ring is built directly onto the 5-phosphoribose-1-pyrophosphate (PRPP) backbone during de novo purine synthesis, and requires the substrates glutamine, glycine, bicarbonate and 10-formyl-THF. De novo purine synthesis is a 10-step cytosolic reaction that results in the production of inosine monophosphate (IMP). IMP is further converted into guanosine monophosphate (GMP) via the activity of the enzymes inosine monophosphate dehydrogenase (IMPDH1) and guanosine monosphosphate synthetase (GMPS), or adenosine monophosphate (AMP) via the activity of the enzyme adenylosuccinate synthase (ADSS) and adenylosuccinate lyase (ADSL). TYMS: thymidylate synthase; dTMP: deoxythymidine monophosphate; SHMT1/2: serine hydroxymethyltrasnferase 1/2; PPAT: phosphoribosyl pyrophosphate amidotransferase; GART: glycinamide ribonucleotide transformylase; MTHFD1L: Methylenetetrahydrofolate Dehydrogenase (NADP+-Dependent) 1 Like; FGAMS: formylglycinamidine ribonucleotide synthase (FGAMS); PAICS: phosphoribosylaminoimidazole carboxylase; AICAR: 5-Aminoimidazole carboxamide ribonucleotide; ATIC: 5-aminoimidazole-4-carboxamide ribonucleotide formyltransferase/IMP cyclohydrolase; XMP: xanthosine monophosphate.

THF, and subsequently 10-formyl-THF, are essential to the synthesis of purine nucleotides [52,53]. Due to the dependency of de novo purine synthesis on 1-C metabolism, deficiencies in 1-C metabolism leading to reduction in its products would result in a lower availability of essential intermediates for purine synthesis. 1-C metabolism also produces other metabolically significant compounds, including glycine and serine. Glycine is a substrate for glutathione and purine synthesis, and serine can be used to synthesize glycine in the absence of an exogenous supply [2,55,56]. 1-C metabolism is compartmentalized between the cytosol and mitochondria. The compartmentalization of these reactions allows for the existence of parallel metabolic processes, increasing the metabolic adaptability of cells [48]. Figure 1 shows a schematic of 1-C-mediated purine synthesis and the enzymes involved in this process.

## 4. 1-C-Mediated de Novo Purine Synthesis: Relevance in Cancer and Glioblastoma

Differential expression of metabolic enzymes, for example, those of glycolysis and the PPP, has been found to be a source of intratumoural heterogeneity in glioblastoma [13], and often results in differential rates of nucleotide synthesis within glioma cells [13]. The enzymes of the mitochondrial folate cycle, including MTHFD2/L and serine hydroxymethyltrasnferase (SHMT), have been found to be expressed at markedly higher levels in cancer cells, including hepatocellular carcinoma, colorectal cancer, breast cancer, and glioblastoma [55,56,57,58,59]. BTICs show increased expression of 1-C metabolism enzymes, and it has been hypothesized that folate cycle reprogramming is associated with acquisition of the stem-like phenotype in glioblastoma tumour cells [31,60]. Alterations in 1-C metabolism have been shown to influence overall survival in some cancers, including head and neck squamous cell carcinomas, colorectal cancer, pancreatic cancer, breast cancer, lung adenocarcinoma, and paediatric medulloblastoma [59,61,62,63,64,65]. Knockdown of MTHFD2/L has been shown to result in reduced cell growth and Ki67 staining, reduced in vivo tumourigenesis, and G0/G1 cell cycle arrest in lung adenocarcinoma [50,66]. Deficiency of MTHFD2/L and alteration of mitochondrial 1-C metabolism result in defects in other metabolic pathways, particularly de novo purine synthesis. Additionally, accumulation of glutaminolysis, glycolysis, and PPP intermediates has been observed after MTHFD2/L knockdown [66]. The inhibition of MTHFD2/L from 1-C metabolism results in purine nucleotide deficiency and reduced cell proliferative capacity, which can be restored by external supplementation of hypoxanthine and the purine salvage pathway [67,68,69]. Studies have shown that knockdown of MTHFD2/L results in reduced rates of IMP, AMP, and GMP—i.e., of the products of de novo purine synthesis [50].

As mentioned previously, purine synthesis is a limiting factor for the growth, proliferation, and maintenance of BTICs [32,70]. Deficiencies in purine synthesis enzymes such as 5-aminoimidazole-4-carboxamide ribonucleotide formyltransferase/IMP cyclohydrolase (ATIC), formylglycinamidine ribonucleotide synthase (FGAMS), adenylosuccinate lyase (ADSL), phosphoribosylaminoimidazole carboxylase (PAICS), guanosine monosphosphate (GMPS) and inosine monophosphate dehydrogenase (IMPDH2) have been found to result in altered purinosome assembly and reduced purine synthesis rates [71,72]. Purine synthesis enzymes are found to be overexpressed in patient populations across a variety of tumour types, including glioblastomas [33,61,73]. Goswami et al. report increased expression of PAICS and PPAT in lung cancer [74]. Expression of PPAT and PAICS was independently associated with patient survival in lung adenocarcinomas; further, a subset of adenocarcinoma patients harbour aneuploidy and amplification in divergently transcribed loci of PPAT and PAICS [74].

Mutations in ADSL are known to abrogate purinosome formation, limiting purine synthesis [50,71]. Purinosome formation is significantly affected in patients with ADSL deficiency, an autosomal recessive disorder of purine metabolism [71]. Skin fibroblasts derived from patients with ADSL deficiency show reduced spatial overlap between the purine synthesis enzymes ADSL, ATIC, GART, and phosphoribosyl pyrophosphate amidotransferase (PPAT), suggesting reduced purinosome formation and reduced purine synthesis [71]. Disruption of purinosome assembly has also been shown to enhance sensitivity to chemotherapy agents such as methotrexate [73]. shRNA-mediated knockdown of ADSL and GMPS in BTICs results in abrogation of self-renewal and tumourigenesis in xenografts [32]. IMPDH2 expression has also been found to be necessary for glioblastoma tumourigenesis in vivo [75]. Knockdown of ADSL and GMPS results in increased levels of cleaved caspase-3 and reduced levels of Ki-67 and SOX2 in BTICs [32]. Additionally, data from The Cancer Genome Atlas (TCGA) show increased expression of PRPS1, GMPS, and ADSL protein in BTICs compared to normal brain tissues [32,76]. Wang et al. show that BTICs have an upregulation of H3K27ac at purine synthesis pathway genes, suggesting priming of purine pathway genes in glioblastoma compared to normal brain tissue [32]. Increased levels of ADSL, adenylosuccinate synthase (ADSS), IMPDH1, and PPAT are associated with poor prognosis in glioblastoma patients [32]. Additionally, overexpression of PPAT, IMPDH1, and ADSS correlate with worse survival among glioblastoma patients [32].

In addition to proliferation of BTICs, purine nucleotide synthesis has been shown to regulate DNA repair and therapeutic resistance in glioblastoma [77]. Overexpression of IMPHD2 in glioblastoma tumour cells results in a high turnover of GTP, which is required for DNA replication and proliferation, rRNA and tRNA synthesis, as well as certain signalling pathways [75,78]. In addition to GTP, extracellular ATP and ADP show extremely low degradation rates in glioma cell lines compared to normal astrocytes, which speaks to the importance of adenosine for glioma cell proliferation [79]. Furthermore, adenosine has neuroprotective abilities that can induce angiogenesis, which makes high adenosine levels even more beneficial to glioma cells [79,80,81]. Downregulation of inosinates and guanilates correlates positively with sensitivity to radiotherapy [77]; while nucleotide availability did not prevent DNA damage induction, exogenous supplementation of purines following treatment with radiation did reduce DNA damage, suggesting that purine nucleotides enhance the ability of glioblastoma cells to repair DNA lesions [77]. Inhibition of GTP synthesis resulted in a reversal of radiotherapy resistance in a patient-derived xenograft (PDX) model of glioblastoma [77]. Other groups suggest that purine synthesis may also be a driver for chemoresistance in glioblastoma cells [78]. TMZ therapy has been shown to results in epigenetic modifications that cause glioblastoma cells to rely on de novo purine synthesis [78]. Increased rates of de novo nucleotide synthesis provide tumour cells with enhanced ability to repair DNA damage caused by alkylating agents, such as TMZ, in addition to preventing cells from recycling damaged nucleotides from the extracellular environment through the purine salvage pathway [78].

The expression of purine synthesis enzymes in glioma initiating cells has been shown to be regulated in a concerted manner, which suggests the influence of upstream transcriptional regulators or programs [32]. Although alteration of purine metabolism has not been exclusively associated with specific oncogenic events in cancer, many oncogenic alterations that drive glioblastoma formation, including of PTEN, EGFR, and PI3CA, can cause similar alternations in nucleotide synthesis and metabolism [67,77,82,83,84]. Table 1 provides a summary of the discussed 1-C metabolism and purine synthesis associated vulnerabilities.

## 5. Signalling Pathways Upstream of Metabolic Reprogramming

A number of signalling pathways have been proposed to be upstream of the metabolic changes described above. The activation of the PI3K/Akt pathway induces excessive glucose uptake and dependency on aerobic glycolysis, while overexpression of Myc can induce uptake of glutamine in excess of bioenergetic needs [15]. The PI3K-Akt and Myc pathways have been associated with increased proliferation and metabolic reprogramming in cancer cells [8,58], as well as regulation of purine synthesis in glioblastoma cells [32]. PI3K-Akt activation has been shown to lead to excessive glucose uptake by cancer cells, increasing their dependence on aerobic glycolysis, and as a consequence increasing the availability of glycolysis intermediates required for biosynthetic pathways [15].

As a master regulator of metabolism, mTORC1 has been studied extensively in the context of cancer cell metabolism, and mTORC inhibitors such as rapamycin have been used to delay tumourigenesis [49]. Activation of the mTORC1-ATF4 axis by growth signals has been shown to lead to an increase in the transcription of MTHFD2/L [2]. Ben-Sahara et al. show that rapamycin-mediated mTORC inhibition results in the depletion of MTHFD2/L, as well as the downstream de novo purine synthesis pathway [67]. Nucleotide metabolism has been reported to be regulated both by oncogenes and tumour suppressors [87]. For example, Mtp53 regulates nucleotide pools by transcriptionally upregulating nucleotide biosynthesis pathways and has been shown to support invasion and proliferation in cancer cell lines [87]. It has also been shown that p53 silencing results in the reduced expression of nucleotide metabolism enzymes, including DHFR, TYMS, and IMPDH1/2 [87].

One of the pathways most extensively studied in relation to purine synthesis regulation is the AMPK signalling pathway. AMPK acts as a metabolic checkpoint regulator of cell growth [6,88]. AMPK is known to be highly active in high-grade gliomas, regardless of their genetic background, and AMPK-mediated transcriptional regulation of bioergenetics has been found to be essential for tumour growth [89,90,91]. While AMPK is more classically known as a suppressor of cell growth due to its inhibitory effects on anabolism, some studies have shown that AMPK-deficient cells are at a growth disadvantage [90,92]. The differential effects of AMPK activation on metabolic reprogramming and growth may be due to the differential environmental stressors impacting cancer cells and the need to adapt to these conditions for survival. For example, AMPK activation can lead to the reduced activity of phosphoribosylpyrophosphate synthetase (PRPS), which is required for the production of the phosphoribosyl backbone of nucleotides via the PPP, a critical substrate for cell replication [50,86,93,94]. Furthermore, AMPK activation has been shown to lead to the sequestration of the de novo purine synthesis enzyme FGAMS [95], which can impair purinosome assembly [52,53,54,71].

While growth signalling pathways can result in metabolic reprogramming of cancer cells, metabolic changes can consequently alter cell signalling pathways. As an example, decreased rate of de novo purine synthesis has been shown to result in accumulation of 5-aminoimidazole carboxamide ribonucleotide (AICAR), the final purine synthesis intermediate before IMP in the de novo purine synthesis pathway [32,66]. AICAR is an activator of AMPK signalling and hence can inhibit cell growth. AICAR treatment results in reduced cell growth in a dose-dependent manner and, combined with gefitinib, has resulted in enhanced sensitivity to the EGFR inhibitor in lung cancer cells [66]. Guo et al. show that AICAR-mediated AMPK activation also leads to negative regulation of glioblastoma cell growth, particularly in EGFR-activated cells [6]. This growth inhibitory effect seems to be mediated through metabolic reprograming, as AICAR treatment resulted in AMPK-mediated inhibition of lipogenesis in EGFR-activated tumours, which could be reversed by exogenous supplementation of malonate and palmitate [6].

## 6. Treatments Targeting 1-C Metabolism and Purine Synthesis in Cancer

Although 1-C-mediated purine synthesis has received significant attention as a regulator of cancer cell proliferation and treatment resistance, the importance of this process as a viable target for anticancer therapy remains understudied [96]. Drugs targeting cytosolic 1-C metabolism, such as methotrexate (MTX) and pemetrexed, have been used as anticancer agents [96]. MTX is a competitive inhibitor of DHFR, while pemetrexed targets multiple enzymes involved in nucleotide synthesis, including DHFR, thymidylate synthase (TYMS), and glycinamide ribonucleotide transformylase (GART) [93,94,97,98]. Walling provides a thorough review of antifolates and their use as therapeutic agents [99]. While these compounds are inhibitors of 1-C metabolism, physiologically relevant concentrations of extracellular hypoxanthine inhibit the toxic effect of MTX, which suggests that MTX-mediated DHFR inhibition also results in downstream inhibition of the purine synthesis pathway [68]. This finding suggests that purine synthesis may also be a viable therapeutic target in cancer.

Drugs that directly inhibit de novo purine synthesis, such as L-alanosine and thiopurines, have also been studied in cancer. The toxicity of these chemicals can be influenced by the expression of other metabolic enzymes or the selective reliance of cancer cells on certain metabolic pathways. For example, sensitivity to thiopurines such as 6-mercaptopurine (6-MP) and 6-thioguanine (6-TG)—compounds extensively used for the treatment of leukaemias—has been shown to be dependent on the expression of methyladenosine phosphorylase (MTAP) [69]. The deletion of the MTAP gene is a frequent event in many cancers, and results in the dependence of cancer cells on de novo purine synthesis or exogenous purine salvage [69,100,101,102,103,104,105]. In the event of limited exogenous purine availability, MTAP-deficient cancer cells are more sensitive to inhibitors of de novo purine synthesis [69,106]. Loss of MTAP in glioblastoma cells promotes stemness as well as susceptibility to purine starvation and inhibition of de novo purine synthesis using L-alanosine [106]. Direct inhibition of purine synthesis in glioblastoma has gained recent therapeutic interest with studies showing the correlation between treatment resistance and purine metabolism in glioblastoma [77,78]. Mycophenolate mofetil (MMF), an inhibitor of IMPDH1 and GTP synthesis, was found to sensitize glioblastoma cells to radiation therapy and significantly improve survival in combination with TMZ in a PDX model of glioblastoma [77,78]. There is currently an ongoing phase 0/I trial of MMF in recurrent and primary glioblastomas (NCT04477200) [107].

One of the major downfalls of targeting metabolic programs in cancer treatments is the possibility of adverse effects that may rise due to disturbance of normal cell metabolism. For example, combination of high-dose MTX with other therapeutic strategies, such as radiotherapy, has shown to result in neurotoxic adverse events [99]. Studies have shown that the same antiproliferative effects observed in cancer cells are not observed in normal cells with the inhibition of mitochondrial 1-C metabolism enzymes [58]. This effect may be due to the existence of parallel 1-C metabolism pathways in the cytoplasm [65] or some toxic event that is unrelated to normal cell metabolism, perhaps related pathways that are further upregulated in highly proliferative cancer cells, such as de novo purine synthesis. Asai et al. have identified chemical compounds, MTHFD2 Inhibitor for THF pocket (MIT) and MTHFD2 Inhibitor for NAD pocket (MIN), that can effectively target and inhibit MTHFD2 in colorectal cancer cells [108]. Additionally, small-molecule inhibitors of SHMT1/2 have been demonstrated to be effective at exerting cytotoxic effects against diffuse B-cell lymphoma progression in vitro [109]. Although both cytosolic and mitochondrial processes are significant for 1-C metabolism, it has been indicated that mitochondrial folate metabolism affects the prognosis of patients more significantly [58,108]. Inhibitors of mitochondrial 1-C metabolism have not been studied in clinical settings; however, pre-clinical studies highlight them as attractive therapeutic targets. This warrants further research into the metabolic reprogramming of 1-C metabolism in cancer cells. Zhou et al. also show that GTP synthesis is preferentially upregulated in glioblastoma cells and not normal brain tissue, resulting in minimal toxic effects of GTP synthesis inhibition in normal cells [77]. In addition to selective targeting of cancer cells, inhibitors of purine synthesis do not require a specific oncogenic event for activity; this means that even genetically heterogeneous tumours can potentially benefit from purine synthesis inhibition [70,78,85]. Table 2 provides a summary of recent studies showing the efficacy of targeting 1-C-mediated purine synthesis enzymes in inhibition of glioblastoma cell growth and tumourigenesis.

## 7. Conclusions

Macromolecules, including nucleic acids, lipids, and proteins, are fundamental requisite substrates for proliferation in all mammalian cells. Cancer cells and stem cells rely on diverse metabolic strategies to maintain macromolecule synthesis. As discussed in this review, a number of 1-C metabolism and purine synthesis-related vulnerabilities exist in glioblastoma cells that can be leveraged to inhibit tumour cell proliferation and tumour growth. To sustain proliferation, glioblastoma cells, and particularly BTICs, upregulate and rely on anabolic pathways such as 1-C-mediated purine synthesis. Multiple studies have suggested that these metabolic vulnerabilities are not associated with specific oncogenic events or specific genetic subtypes in glioblastoma, yet are specific to tumour cells. As a result, tumour-specific 1-C-mediated purine synthesis vulnerabilities may be effective therapeutic targets to inhibit tumour growth with minimal adverse effects on normal cells. The importance of nucleotide synthesis pathways for maintenance of BTICs also suggests that these metabolic pathways may offer an attractive strategy to overcome treatment resistance and prevent tumour recurrence. Further research is required to understand the underlying mechanisms through which these vulnerabilities may arise in BTICs. Such studies can elucidate more concrete ways to target the metabolic processes that underly the glioma proliferation and resistance.

## Figures and Tables

**Table 1:** Summary of described 1-C metabolism and purine synthesis associated vulnerabilities in cancer.

Metabolic Enzyme	Implication	Cancer Type/Cell Type	Reference
MTHFD2	Cell growth and tumourigenesis; knockdown of MTHFD2 resulted in reduced cell growth and Ki-67 staining	Lung adenocarcinoma	[66]
MTHFD2	Cell migration and invasion; overexpression associated with poor prognosis and increased metastasis	Breast cancer	[85]
MTHFD2	Cell growth and survival; metabolic adaptation to glutamine starvation	Glioblastoma	[86]
DHFR, SHMT1, MTHFD1	Tumour sphere formation, methionine dependency, and stem-like phenotype	Glioblastoma	[31]
MTHFD2	Highly overexpressed; overexpression associated with poor prognosis	Various cancer types	[58]
SHMT2	Polymorphisms associated with increased risk of cancer	Squamous cell carcinoma of the head and neck	[61]
SHMT2, MTHFD2, MTHFD1	Overexpressed and associated with increased proliferation; associated with increased mortality in breast cancer	Various cancer types	[56]
MTHFD2, SHMT2, ALDH1L2 ^1^	Overexpressed; overexpression associated with poor prognosis	Colorectal cancer	[59]
MTHFD2 and SHMT2, ALDH1L2	High expression associated with lower overall survival and shorter progression free survival	Pancreatic cancer	[62]
DHFR, TYMS, MTHFD2	Overexpression associated with poor prognosis	Group 4 Medulloblastoma	[63]
PPAT, PAICS	Overexpressed; overexpression associated with aneuploidy and gene amplification in subgroup of patients	Lung adenocarcinoma	[74]
DHFR, TYMS, MTHFD2	Tumourigenesis; overexpression associated with poor prognosis	Brain tumour initiating cells	[32]
IMPDH2	Cell proliferation and tumourigenesis; overexpression associated with poor prognosis	Glioblastoma	[75]
IMPDH2	Chemoresistance	Glioblastoma	[78]
IMPDH2	Resistant to radiotherapy	Glioblastoma	[77]

^1^ ALDH12L: aldehyde dehydrogenase 1 family member L 2.

**Table 2:** Summary of recent studies targeting 1-C metabolism and purine synthesis-related metabolic pathways in glioblastoma.

Chemical Compound/Drug	Metabolic Target	Reference
Mycophenolate Mofetil	IMPDH2	[107]
Mycophenolate Mofetil	IMPDH2; Purine synthesis	[78]
Mycophenolate Mofetil	IMPDH2; Purine synthesis	[77]
Methotrexate	DHFR; Folate-mediated 1-C metabolism	[60]
Pemetrexed	DHFR, TYMS; Folate-mediated 1-C metabolism, nucleotide synthesis	[110]
siRNA-mediated knockdown SERBP1 ^1^	SERBP1 Methionine synthesis and 1-C metabolism	[111]
siRNA-mediated knockdown of MTHFD2	MTHFD2; Purine synthesis	[86]
L-Alanosine	ADSS; Purine synthesis	[106]
Adenosine Deaminase	Adenosine synthesis	[112]
shRNA-mediated knockdown of PRPS1 ^2^, GMPS and ADSL	De novo purine synthesis enzymes	[32]

^1^ SERBP1: Serpine1 mRNA-binding protein; ^2^ PRPS1: phosphoribosyl pyrophosphate synthetase.

## Data Availability

Not applicable.
